# Red Light-Emitting Diodes with All-Inorganic CsPbI_3_/TOPO Composite Nanowires Color Conversion Films

**DOI:** 10.1186/s11671-020-03430-w

**Published:** 2020-11-16

**Authors:** Lung-Chien Chen, Yi-Tsung Chang, Ching-Ho Tien, Yu-Chun Yeh, Zong-Liang Tseng, Kuan-Lin Lee, Hao-Chung Kuo

**Affiliations:** 1 Department of Physics, School of Science, JiMei University, Xiamen, 361021 China; 2 Department of Electro-Optical Engineering, National Taipei University of Technology, Taipei, 10608 Taiwan; 3 Department of Electronic Engineering, Ming Chi University of Technology, New Taipei City, 24301 Taiwan; 4 Department of Photonics and Institute of Electro-Optical Engineering, National Chiao Tung University, Hsinchu, 30010 Taiwan

**Keywords:** Perovskite quantum dot, CsPbI_3_, Color conversion film, Trioctylphosphine oxide

## Abstract

This work presents a method for obtaining a color-converted red light source through a combination of a blue GaN light-emitting diode and a red fluorescent color conversion film of a perovskite CsPbI_3_/TOPO composite. High-quality CsPbI_3_ quantum dots (QDs) were prepared using the hot-injection method. The colloidal QD solutions were mixed with different ratios of trioctylphosphine oxide (TOPO) to form nanowires. The color conversion films prepared by the mixed ultraviolet resin and colloidal solutions were coated on blue LEDs. The optical and electrical properties of the devices were measured and analyzed at an injection current of 50 mA; it was observed that the strongest red light intensity was 93.1 cd/m^2^ and the external quantum efficiency was 5.7% at a wavelength of approximately 708 nm when CsPbI_3_/TOPO was 1:0.35.

## Background

Numerous types of quantum dots (QDs), including CdSe QDs [1], carbon QDs [2], InP QDs [3], CuInS_2_ QDs [4], CdTe QDs [5], and perovskite QDs [6, 7], were widely studied to be involved in the main mechanism that underlies the observed phenomenon. QDs have been utilized in the field of light-emitting diodes (LEDs) [8, 9], solar cells [10, 11], photodetectors [12, 13], and bio-markers [14, 15] and have been adopted to construct sensors to detect biologically interesting molecules [16]. In particular, a perovskite material was the most popular potential materials in recent years, and enormous progress and applications have been made in this direction [17–23]. They can be synthesized to have various dimensional morphologies, including three-dimensional (3D) morphologies such as thin film and bulk single crystal, two-dimensional (2D) morphologies such as nanoplates and nanosheets, one-dimensional (1D) such as nanowires and nanorods, and zero-dimensional (0D) morphologies such as QDs and nanoparticle structures. All-inorganic perovskite QDs (CsPbX_3_, *X* = Cl, Br, I) have excellent optical properties such as a high absorption coefficient, a narrow half-peak width of 20–40 nm, a quantum yield of up to 90%, and higher stability than hybrid organic–inorganic perovskite QDs [such as MAPbX_3_ and FAPbX_3_ (*X* = Cl, Br, I)] [24–27]. The synthesis method is simple and low cost and is expected to replace traditional fluorescent materials. Moreover, by adjusting the ratio of halogen element X (*X* = Cl, Br, I), we can adjust the emission wavelength of perovskite CsPbX_3_ QDs from 380 to 780 nm and can achieve an all-visible light region [28–30]. The integration of perovskite QDs into LEDs can achieve a breakthrough of more than 110% of the NTSC color gamut and a better color rendering performance [23, 31–34]. This showed that CsPbI_3_ QD has considerable potential to become a candidate material for red phosphor. In contrast, cadmium-containing QDs were highly toxic. After they were prepared into various types of application-end products, the environmental damage was considerable. Considering environmental protection issues, the development of cadmium-free QD materials is necessary, but the efficiency of cadmium-free materials is poor, the full width at half maximum (FWHM) is wide, the improvement in efficiency and the control of FWHM are the focus of the development of cadmium-free QDs, and the instability of perovskite-based devices still hinders their entry to the commercial market [35]. As far as we know, there have been few reports on the use of CsPbI_3_ QDs as red phosphor to manufacture red LEDs, most of which include the addition of the halogen element Br to form CsPbBr_*x*_I_3−*x*_ QDs [36–38].

Trioctylphosphine oxide (TOPO), a highly branched capping ligand with a strong steric effect, is commonly used as a capping ligand for conventional II–VI, III–V, and IV–VI QDs [39–41]. Because of the highly branched molecular structure and the relatively strong coordination ability of the P=O group, TOPO species can cooperate with the surface of the obtained QDs through a certain scheme, thereby providing a more complete surface passivation for the QDs [42–44]. Zhang and co-workers successfully synthesized the monodisperse TOPO-capped CsPbX_3_ QDs with excellent stability against an ethanol solvent attack by introducing TOPO in the Pb precursor with an oleic acid (OA) and oleylamine (OAm) system [45]. Zhang et al. [46] performed a novel synthesis of CsPb_*x*_Mn_1−*x*_Cl_3_ QDs by using TOPO and a Mn organometallic complex as the Mn reaction precursor, which exhibited PLQYs as high as 63% and excellent dispersibility and stability. Herein, we present a hot-injection method to synthesize CsPbI_3_ QDs and then prepare a perovskite CsPbI_3_/TOPO composite with high PL intensity by introducing TOPO into the CsPbI_3_ QD solution. We found that the CsPbI_3_/TOPO composite could form CsPbI_3_ nanowires and QDs, as well as show excellent material and optical characteristics. Then, the CsPbI_3_/TOPO composite was uniformly mixed with UV resin to prepare a color conversion fluorescent film, and a color-converted pure red LED was obtained by exciting the blue GaN-based LED chip.

## Methods

Cesium carbonate (Cs_2_CO_3_, 99.998%) and lead (II) iodide (PbI_2_, 99.999%) were purchased from Alfa Aesar. 1-octadecene (ODE, 90%), oleic acid (OA, 90%), oleylamine (OAM, 90%), and trioctylphosphine oxide (TOPO, 99%) were purchased from Sigma-Aldrich. Ethyl acetate (EA), n-hexane, and acetone were purchased from Echo Chemical. Ultraviolet (UV) resin (U-76063S-A) was purchased from Synergy Innovation.

**Fig. 1:**
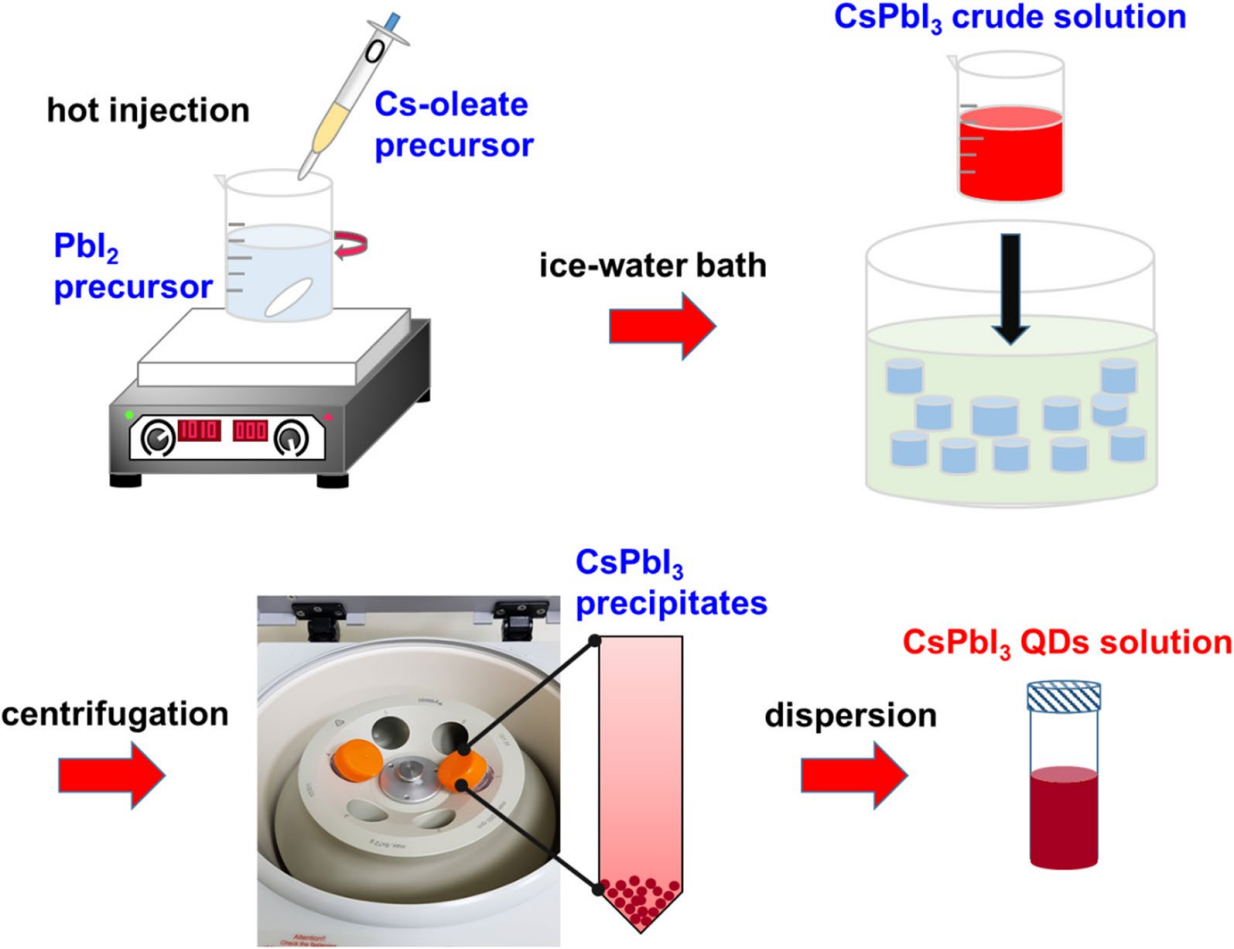
Schematic representation of the synthesis of perovskite CsPbI_3_ QDs via hot-injection and ice water bath methods

Perovskite CsPbI_3_ QDs were prepared by using the hot-injection and ice water bath methods, as presented in Fig. 1. Firstly, 81.4 mg of Cs_2_CO_3_ and 0.25 mL of OA were added to a glass vial containing 3 mL of ODE, and the mixture was placed on a 200 °C hot plate and stirred magnetically for 0.5 h until completely dissolved to form an optically clear Cs-oleate precursor solution. Then, PbI_2_ (200 mg), OA (1 mL), and OAm (1 mL) were added to a glass bottle containing ODE (10 mL), and the mixture was placed in a 140 °C heating bag and stirred for 0.5 h until the PbI_2_ salt had completely dissolved. Thereafter, the heating temperature was increased to 160 °C and stirred for 5 min, followed by quickly injecting 0.8 mL of the Cs-oleate precursor solution by using a micro-dropper. After 10 s, the CsPbI_3_ crude solution was placed in an ice water bath for 40 s to immediately stop the reaction and was cooled to room temperature. To wash the CsPbI_3_ QDs, the crude solution was precipitated by using the EA washing solvent in a volume ratio of 1:4 via centrifugation with 6000 rpm for 15 min and finally dispersed in 1 mL of n-hexane under ultrasonication for further use. All the synthesis and washing occurred under ambient atmospheric conditions.

Furthermore, 20 mg of TOPO powders was added into 1 mL of hexane and at room temperature while stirring at 600 rpm until the powders were completely dissolved. Subsequently, the perovskite CsPbI_3_ QD solution was added to the TOPO/hexane system with different volume ratios (volume ratios 1:0.15, 1:0.35, and 1:0.60 of CsPbI_3_ QDs and TOPO) while stirring for 1 min at room temperature to obtain the CsPbI_3_/TOPO composites.

**Fig. 2:**
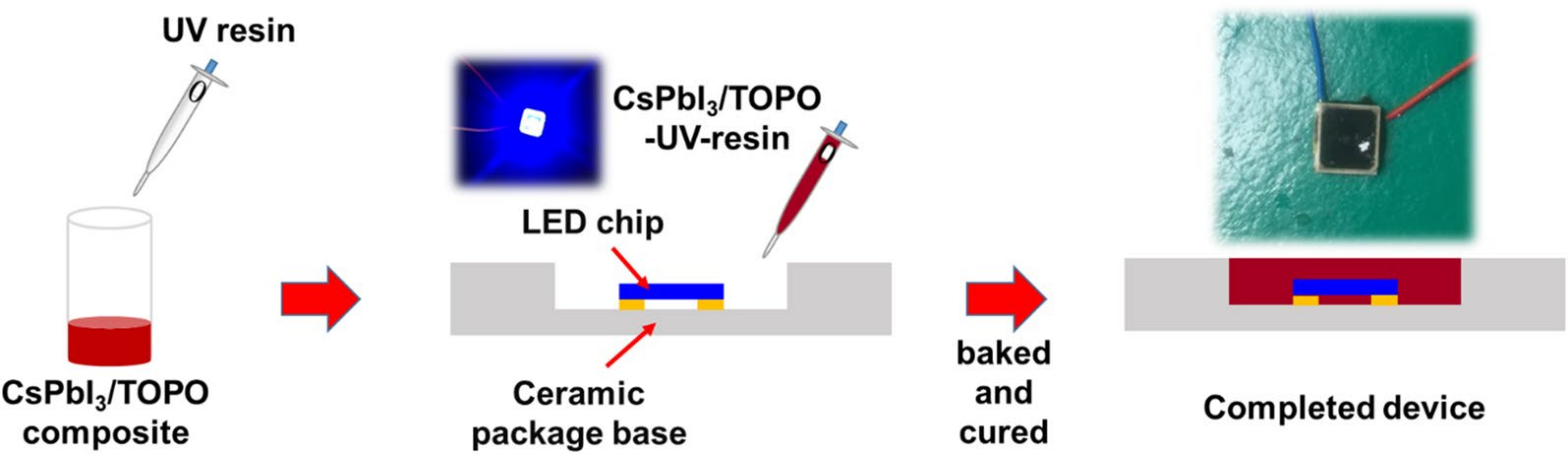
Schematic representation of encapsulating strategy

The different ratios of CsPbI_3_/TOPO composites were mixed with the UV resin (volume ratio 1:2 of CsPbI_3_/TOPO composite and UV resin). Then, the resulting mixture was vacuumed for 0.5 h to remove the bubbles. The different ratios of CsPbI_3_/TOPO–UV resins were obtained. The blue GaN-based LED chip (1 mm × 1 mm) with an emission wavelength of 455 nm was mounted in a groove with a diameter of approximately 7 mm. Thereafter, these mixtures were coated/filled onto glass substrates and blue LED chips and baked at 40 °C for 3 min and then cured using a 365 nm UV lamp for 60 s in the glove box to form color conversion films and color-converted red LEDs, as shown in Fig. 2.

For the characterization, the crystal phases, absorption spectrum, photoluminescence (PL) spectra, and PL quantum yield (PLQY) of CsPbI_3_ QDs and CsPbI_3_/TOPO composites were obtained using field-emission scanning electron microscope (FESEM) (ZEISS Sigma, ZEISS, Munich, Germany), high-resolution transmission electron microscopy (HRTEM) (JEM-2100F, JEOL, Tokyo, Japan), X-ray diffraction (XRD) with CuKα radiation (X’Pert PRO MRD, PANalytical, Almelo, The Netherlands), UV–Vis spectrophotometer (Thermo Scientific™ Evolution 220, Thermo Fisher Scientific, Taiwan), fluorescence spectrophotometer (F-7000, Hitachi, Tokyo, Japan), and a FluoroMax spectrofluorometer with an integrating sphere fiber coupled to a fluorometer (Horiba Jobin Yvon, Longjumeau, France). The current–voltage (I–V), luminance, external quantum efficiency (EQE) characteristics, and electroluminescence (EL) spectra of perovskite color-converted red LEDs were measured by a Keithley 2400 source meter and a Spectrascan^®^ spectroradiometer PR-670 (Photo Research Inc., Syracuse, NY, USA) at room temperature.

## Results and Discussion

**Fig. 3:**
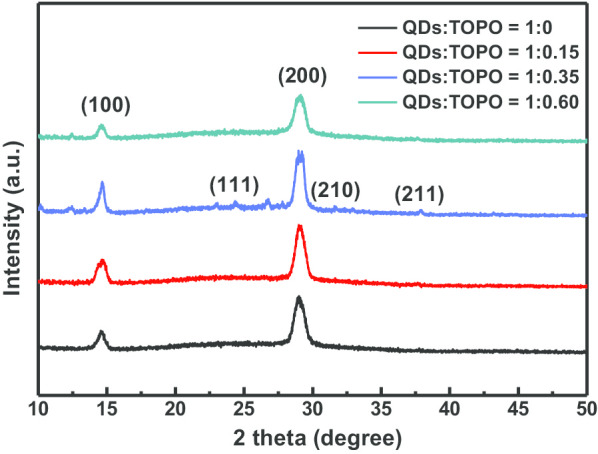
X-ray diffraction (XRD) patterns of CsPbI_3_/TOPO composite films with different ratios

The crystal structures of the obtained CsPbI_3_/TOPO composite films with different ratios were characterized by using XRD, as shown in Fig. 3. The addition of TOPO did not change the microscopic reorganization of CsPbI_3_ QDs, and the QDs were located at approximately 14.95° and 29.1°, corresponding to the (100) and (200) crystal planes of the CsPbI_3_ cubic lattice structure, respectively. Moreover, no crystal binding or by-products appeared with other small crystal diffraction peaks. When the CsPbI_3_/TOPO ratio was 1:0.35, the diffraction peak of the perovskite CsPbI_3_/TOPO composite film in the XRD pattern was stronger and sharper than that of the other CsPbI_3_/TOPO ratios; meanwhile, the (111), (210), and (211) crystal planes of other cubic lattice structures appeared, which confirmed that the perovskite composite prepared with this parameter had better crystallinity [47, 48]. In contrast, excessive TOPO (CsPbI_3_/TOPO = 1:0.60) led to a decrease in perovskite crystallinity, which could be attributed to the excessive amount of TOPO that caused the CsPbI_3_ QDs to produce more nanowire-like structures, resulting in a decrease in film compactness.

**Fig. 4:**
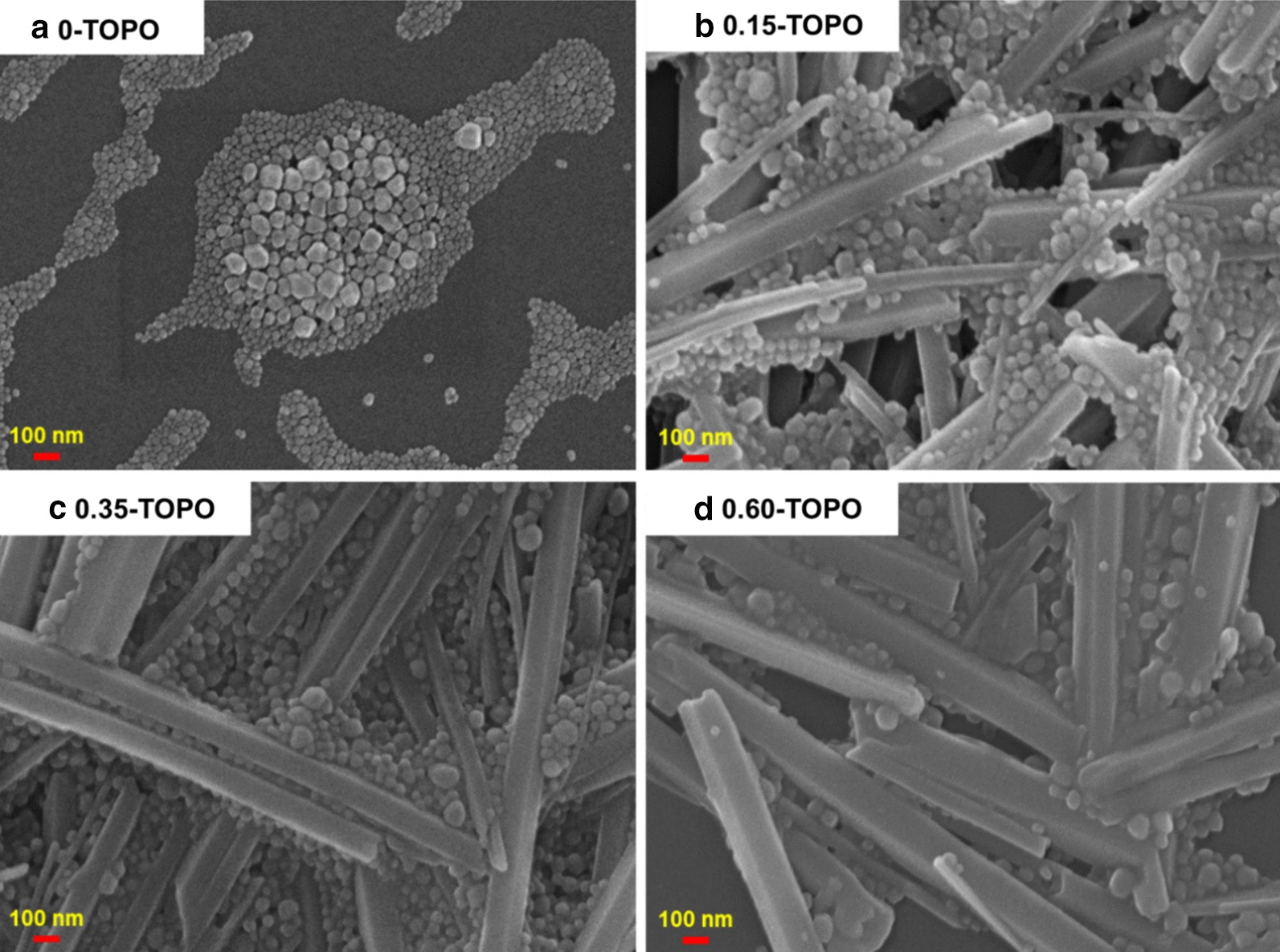
Top view of SEM micrographs of CsPbI_3_/TOPO composite films with different ratios: **a** 1:0, **b** 1: 0.15, **c** 1:0.35, **d** 1:0.60

Figure 4 shows the film formation SEM images of CsPbI_3_/TOPO composite films with different ratios coated on the glass substrates. Figure 4a shows the morphology of the CsPbI_3_ QD film without TOPO, which was formed by the aggregation of discontinuous, large grains and QDs. After the addition of different ratios of TOPO, surprisingly, the nanowires of the CsPbI_3_/TOPO composite films with diameters of 50–160 nm and lengths up to several microns, as well as QDs adhered to the nanowires, were observed (Fig. 4b–d). In addition, when the amount of TOPO increased, most of the CsPbI_3_/TOPO composite materials formed thicker nanowires and the QD grain size increased, resulting in reduced film coverage and poor quality.

**Fig. 5:**
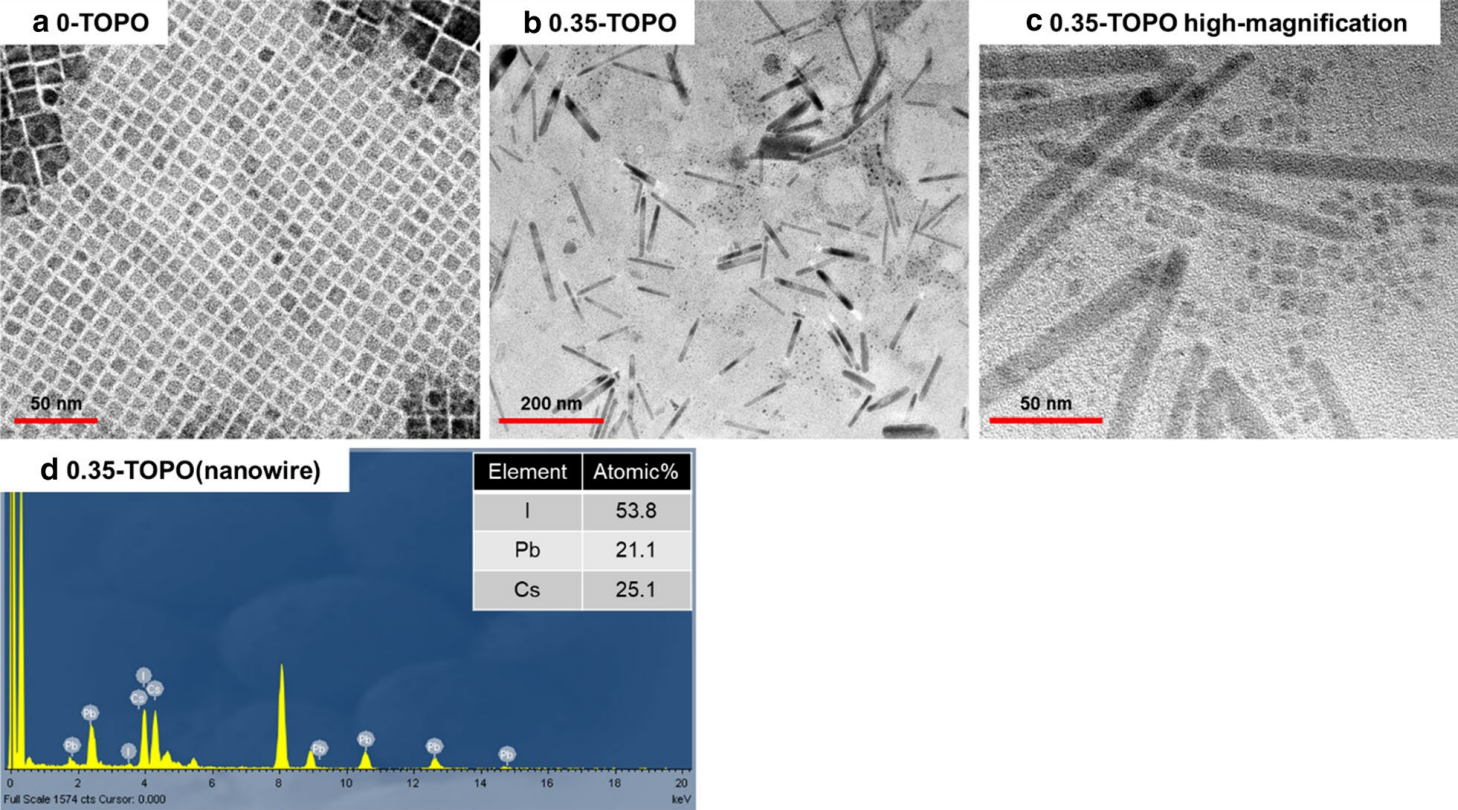
High-resolution TEM (HRTEM) micrographs of CsPbI_3_/TOPO composite solutions with different ratios: **a** 1:0, **b** 1:0.35, **c** 1:0.35 high-magnification, **d** energy-dispersive X-ray (EDX) analysis for the nanowire in the CsPbI_3_/TOPO composite

According to the XRD and SEM results, nanowires and QDs can be obtained by adding TOPO to the CsPbI_3_ QD solution. We chose to have better CsPbI_3_/TOPO composite (CsPbI_3_/TOPO = 1:0.35) quality and analyze its nanowires and QDs by using HRTEM. The HRTEM images of the perovskite CsPbI_3_ QDs and the CsPbI_3_/TOPO composite (CsPbI_3_/TOPO = 1:0.35) solutions are displayed in Fig. 5a, b. Figure 5a clearly shows that TOPO-free CsPbI_3_ had a cubic shape and uniformly arranged QDs and was measured to have a narrow size distribution in the range of 7–12 nm. CsPbI_3_ nanowires and QDs were obtained when the ratio was CsPbI_3_/TOPO = 1:0.35, as shown in Fig. 5b. The nanowires of the CsPbI_3_/TOPO composite were in a broad diameter range of 7–14 nm with a length range of 50–170 nm, and the particle size range of QDs was 5–8 nm (Fig. 5c). We attributed the formation of the nanowire-type structure to the coordination bonds between the O-donor base in TOPO (Lewis base) and the perovskite QDs. It was attributed to the fact that the Pb in the CsPbI_3_ was a Lewis acid and TOPO was a Lewis base. In the Lewis acid–base interactions, a base was defined as the electron donors and an acid was defined as the electron acceptors. A Lewis acid–base reaction occurred when a base donated a pair of electrons to an acid, which formed a Lewis acid–base adduct, a compound that contained a coordinate covalent bond between the Lewis acid and the Lewis base [30, 47]. An energy-dispersive X-ray (EDX) analysis was performed to check the composition and the stoichiometric ratio of the nanowires in the CsPbI_3_/TOPO composite, and the result is shown in Fig. 5d. There were no impurity element-related peaks in the EDX spectrum, which confirmed the XRD result of the pure phase formation. The observed constituent elements and atomic ratios were proved to be CsPbI_3_. In addition, we found that the size of the nanowires and QDs as observed by TEM was different from that obtained from the SEM analysis, which might be attributed to the aggregation phenomenon caused by the solution after spin coating.

**Fig. 6:**
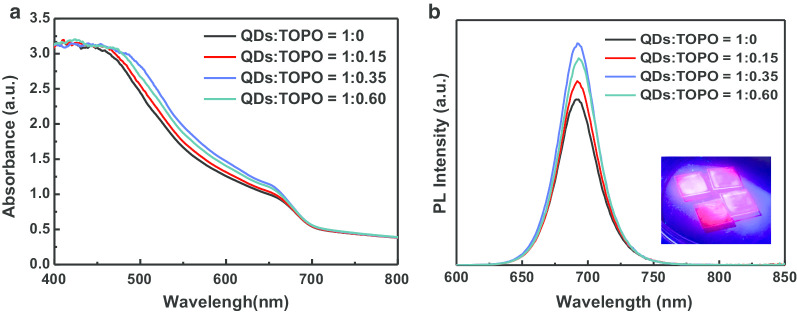
**a** Ultraviolet–visible (UV–Vis) absorption spectra, **b** photoluminescence (PL) spectra of CsPbI_3_/TOPO composite films with different ratios; the inset is the fluorescence photograph of the CsPbI_3_/TOPO composites/glass under 365 nm light excitation

Figure 6 compares the effects of different TOPO ratios on the UV–Vis absorption and PL spectra of the perovskite CsPbI_3_/TOPO composite films, where the absorption peak was at approximately 700 nm, while the PL peak was located at approximately 692 nm. Table 1 shows the optical properties of CsPbI_3_ QDs and CsPbI_3_/TOPO composite films. Figure 6a shows that the TOPO treatment caused a slight shift in absorption; it was observed that the absorption of the CsPbI_3_/TOPO composite film enhanced slightly as the TOPO content increased. However, the absorption slightly declined when the ratio of CsPbI_3_/TOPO exceeded 1:0.35. In the visible-light region (470–800 nm), the absorbance of the CsPbI_3_/TOPO composite film prepared with the CsPbI_3_/TOPO ratio of 1:0.35 increased, indicating improved crystallinity. Figure 6b shows the observation that the PL intensity of all perovskite CsPbI_3_/TOPO composite films added with TOPO was higher than that of the CsPbI_3_ QD film without TOPO. When UV light was irradiated on the perovskite CsPbI_3_/TOPO composite films, the films absorbed the photons and caused the electrons in the valence band to jump to the conduction band. The photons in the conduction band transitioned back to the valence band for emission or to fall into the traps in the film to be quenched. Therefore, when the perovskite CsPbI_3_/TOPO composite films had high quality and relatively few traps or defects, the fluorescent signal was stronger. When the CsPbI_3_/TOPO ratio was 1:0.35, the PL intensity was the strongest with a high PLQY of 47.2% and a narrow FWHM of approximately 36.4 nm, which implied that the perovskite CsPbI_3_/TOPO composite film prepared in this ratio was of high quality.

**Table 1:** Optical properties of CsPbI_3_ QDs and CsPbI_3_/TOPO composite films

QDs/TOPO	1:0	1:0.15	1:0.35	1:0.60
PL (nm)	691	692	692	693
FWHM (nm)	37.9	37.2	36.4	36.8
PLQY (%)	32.8	35.8	47.2	37.4

**Fig. 7:**
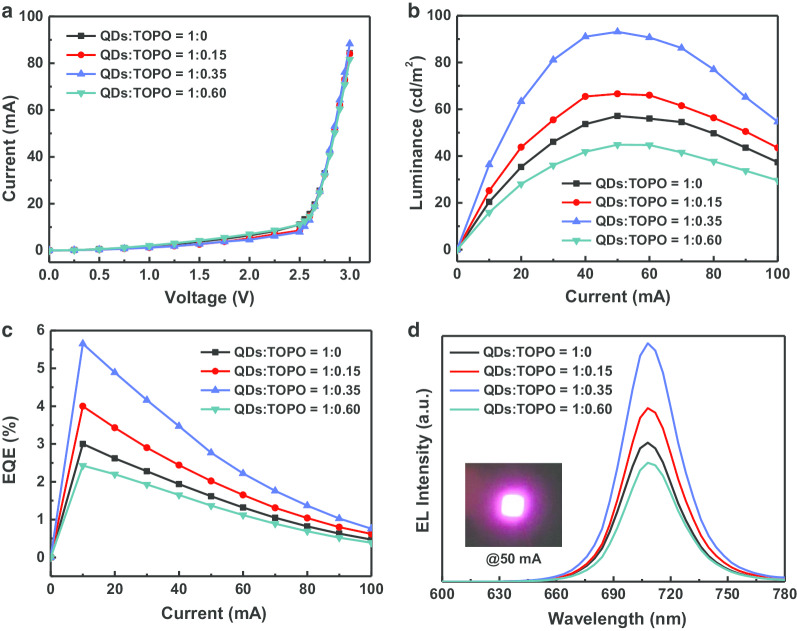
Performances of CsPbI_3_/TOPO composite-converted red LEDs under different driving current. **a** I–V, **b** L–I, **c** EQE–I curves, **d** EL spectra. The inset is an optical photograph of a color-converted red LED at 50 mA

As presented in Fig. 7a, the I–V curves of the CsPbI_3_/TOPO composite-converted red LEDs with different ratios were almost the same, confirming that the coating QDs had nearly no influence on the LED circuit. The luminance–current (L–I) and EQE–current (EQE–I) characteristics for all the LED devices are shown in Fig. 7b, c, and the optoelectronic characteristics of the devices are summarized in Table 2. We found that the maximum brightness and EQE values of the devices increased first and then slightly declined with a continuous increase in the TOPO content of the CsPbI_3_/TOPO composite. The performances of the CsPbI_3_/TOPO composite-converted red LEDs could be optimized by altering the TOPO amount, and the optimized ratio of CsPbI_3_/TOPO was 1:0.35. The optimized CsPbI_3_/TOPO composite-converted red LED device exhibited a turn-on voltage of 2.65 V (@20 mA) and maximum brightness and EQE values of 93.1 cd/m^2^ and 5.7%, respectively, which were significantly better than those of the other devices. In contrast, the maximum brightness and EQE values of the other CsPbI_3_/TOPO ratios (1:0, 1:0.15, and 1:0.60) were 57.1, 66.5, and 44.8 cd/m^2^, as well as 3.0%, 4.0%, and 2.4%, respectively. However, the surface defects caused by the CsPbI_3_/TOPO composite films treated with excessive TOPO content reduced the ability of fluorescence conversion, resulting in a significant decrease in both luminance and EQE. This result was inferred from the SEM observation that excessive TOPO content led to a decrease in the film coverage and quality. The emission spectra of all the CsPbI_3_/TOPO composite-converted red LEDs with different ratios under a driving current of 50 mA are shown in Fig. 7d, which illustrates that all the color-converted devices had a major EL peak at 708 nm with a FWHM of approximately 34 nm.

**Table 2:** Optoelectronic properties of CsPbI_3_/TOPO composite-converted red LEDs

QDs/TOPO	1:0	1:0.15	1:0.35	1:0.60
*L*_max_ (cd/m^2^)	57.1	66.5	93.1	44.8
EQE_max_ (%)	3.0	4.0	5.7	2.4

**Fig. 8:**
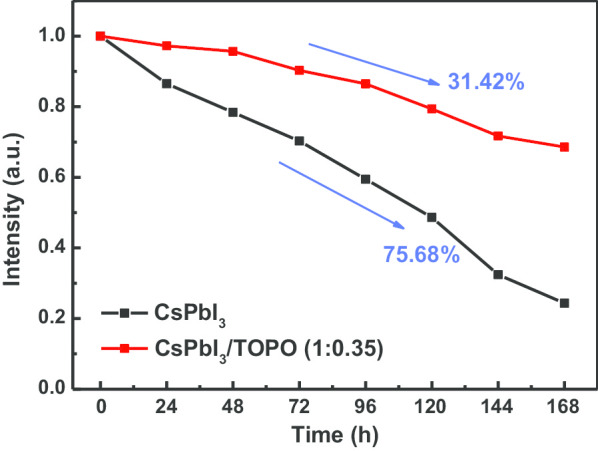
Stability of CsPbI_3_-converted and CsPbI_3_/TOPO composite-converted red LEDs

We found that the luminance of a CsPbI_3_/TOPO composite-converted red LED dropped by only 31.42%, whereas it dropped by up to 75.68% for a CsPbI_3_-converted red LED, as shown in Fig. 8. The luminance of a CsPbI_3_-converted red LED showed a rapid linear decrease with an increase in the stored time, while a CsPbI_3_/TOPO-converted red LED showed that ∼ 85% of the initial value was maintained even within the first four days. Thus, we concluded that a CsPbI_3_/TOPO-converted red LED not only had more luminance than the CsPbI_3_-converted design but also improved stability. Although a CsPbI_3_/TOPO composite material is proposed to incorporate TOPO to improve the quality of the quantum-sized composite material, the stability of the composite material still needs to be improved to meet the practical application standards in future work.

## Conclusions

In conclusion, we presented a simple method to prepare all-inorganic perovskite CsPbI_3_ QDs under ambient atmosphere and then combined a TOPO solution to obtain the CsPbI_3_/TOPO composite including QDs and NWs. The TEM image was obtained; it revealed that the perovskite CsPbI_3_ gradually changed from a QD type to a nanowire type with an increase in the amount of TOPO. The PL spectra were examined. They revealed that the PL intensity of CsPbI_3_/TOPO composites increased with increasing TOPO; the PLQY of the CsPbI_3_/TOPO composite also improved as compared to that of the TOPO-free CsPbI_3_ QDs. Finally, it was applied in a color conversion device using the UV resin; it could be easily made into a quantum composite thin film and affected by water and oxygen, thereby extending the lifetime of the CsPbI_3_/TOPO composite in the atmospheric environment.

## Data Availability

All the data are fully available without restrictions.

## Abbreviations

CsPbI_3_: Cesium lead tri-iodide
Cs_2_CO_3_: Cesium carbonate
PbI_2_: Lead iodide
ODE: Octadecene
OA: Oleic acid
OAM: Oleylamine
EA: Ethyl acetate
TOPO: Trioctylphosphine oxide
QDs: Quantum dots
LED: Light-emitting diode
